# Transgenic Animal Models to Visualize Cancer-Related Cellular Processes by Bioluminescence Imaging

**DOI:** 10.3389/fphar.2019.00235

**Published:** 2019-03-15

**Authors:** Isabella Manni, Luisa de Latouliere, Aymone Gurtner, Giulia Piaggio

**Affiliations:** UOSD SAFU, Department of Research, Diagnosis and Innovative Technologies, IRCCS-Regina Elena National Cancer Institute, Rome, Italy

**Keywords:** cancer, BioLuminescent Imaging (BLI), macroenvironment, animal models, *in vivo* image system

## Abstract

Preclinical animal models are valuable tools to improve treatments of malignant diseases, being an intermediate step of experimentation between cell culture and human clinical trials. Among different animal models frequently used in cancer research are mouse and, more recently, zebrafish models. Indeed, most of the cellular pathways are highly conserved between human, mouse and zebrafish, thus rendering these models very attractive. Recently, several transgenic reporter mice and zebrafishes have been generated in which the luciferase reporter gene are placed under the control of a promoter whose activity is strictly related to specific cancer cellular processes. Other mouse models have been generated by the cDNA luciferase knockin in the locus of a gene whose expression/activity has increased in cancer. Using BioLuminescence Imaging (BLI), we have now the opportunity to spatiotemporal visualize cell behaviors, among which proliferation, apoptosis, migration and immune responses, in any body district in living animal in a time frame process. We provide here a review of the available models to visualized cancer and cancer-associated events in living animals by BLI and as they have been successful in identifying new stages of early tumor progression, new interactions between different tissues and new therapeutic responsiveness.

## Bioluminescence Imaging Technique – Bli

*In vivo* bioluminescence imaging, BLI, represents an interesting current and future new approach to molecular imaging. It allows imaging of internally generated light linked to specific physiological and/or pathological cellular processes in living small animals. This non-invasive technique, allows quantification in the same animal of spatial and temporal progression of the process of interest and identification of animal-to-animal variations (Signore et al., 2010).

**FIGURE 1:**
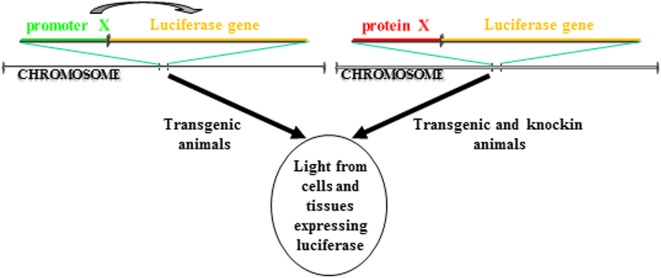
Different strategies to make a luciferase-based transgenic animal. One strategy take advantage of the transgenesis of cassettes in which the luciferase gene transcription is regulated by a promoter. The luciferase will be expressed only in cells in which the promoter is active. In another strategy, the cassette for transgenesis can include a fusion protein between the luciferase and the gene of interest. The cassette can be either knockin into the locus of the gene coding for the protein so that the cassette is under the transcription control of the endogenous promoter or the protein of interest can be directly inserted in frame into the locus. In both cases, the luciferase will be expressed only in cells in which the protein is expressed.

BioLuminescence imaging in living animals takes advantage of luciferase reporter genes as internal sources of light. Usually, using tissue specific promoters, animals are engineered to express luciferase gene in a specific tissue and/or in a particular cellular process. If the activity of the promoter is strictly dependent on a single protein, this approach allows to followed *in vivo* the transcription activity of that specific protein. Another strategy involves the cloning of the luciferase cDNA in the locus of the gene whose expression you want to follow in time. In contrast to the above startegy, in this case is visualized the expression and not the activity of the protein of interest. Both these strategies enable real-time non-invasive imaging of several biological processes (Figure 1). In addition, with the advent of CRISPR/Cas technology, it will be possible from now to insert the luciferase gene in the locus of interest in a manner much easier than in the past. Until now, the animal model that has been most used to study via BLI cellular processes is the mouse model, however, zebrafish models for BLI have been recently described, too.

**FIGURE 2:**
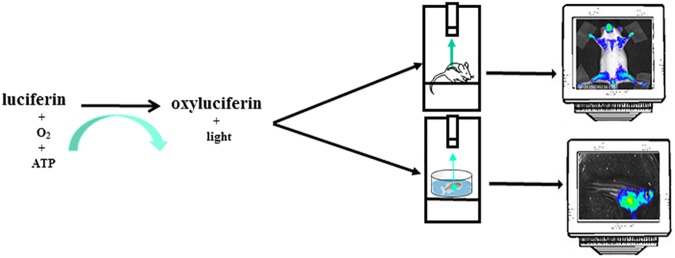
The instrument used for luminescence is composed of an ultra-sensitive cooled CCD camera linked to a dark box. In the dark box is present a shelf heated at 37°C where the mice, or the petri dish with zebrafishes, are positioned. The mice are euthanized trough a system delivering isoflurane or by i.p. injection of sedatives. For zebrafish imaging sessions the sedative is dissolved in the water. The CCD camera detects light coming from mice or zebrafishes. The images are collected and analyzed by a dedicated software. The mice image is referred to Mito-Luc model described in the text (Goeman et al., 2012). The zebrafish image represents a not already published bioluminescent model of proliferation (GP personal communication).

The most common reporter gene useful for BLI is the firefly (*Photinus pyralis*) luciferase, a heat-unstable enzyme with a half-life of approximately 2h thus useful to study dynamics of biological processes. Animals do not produce the substrate for the light producing process, the luciferin, thus giving an excellent signal-to-noise ratio, as there is virtually no background in the animal tissues. Upon intraperitoneal (i.p.) injection the luciferin distributes throughout the mouse rapidly and it passes across blood-tissue barriers including placenta (Lipshutz et al., 2001; Ciana et al., 2003). Likewise in mice, in zebrafish models, luciferin can be injected intraperitoneally or simply dissolved in aquarium water, letting the fish swimming (Chen et al., 2013). In organs expressing luciferase and in the presence of oxygen and ATP as a source of energy, the luciferin become oxyluciferin by a reaction catalyzed by luciferase and coupled with light emission (Figure 2). In addition to the firefly, many other luciferases are available for application of *in vivo* BLI. Among them, the most widely used are the sea pansy Renilla reniformis, the click beetle Pyrophorus plagiophthalamus, the marine copepod Gaussia princeps and the recently developed deep-sea shrimp derived NanoLuc. The substrate for the first two is d-luciferin while for Gaussia is coelenterazine and for NanoLuc a novel coelenterazine analog, furimazine (for a detailed review on available luciferase genes see Mezzanotte et al., 2017). The animals are imaged with cooled charge-coupled device (CCD) cameras mounted within a light-tight box in which the anesthetized animals are placed. The camera is accompanied with computer software for image data acquisition and analysis. The software converts electron signals into numerical values. The data are quantified by region-of-interest analysis, measuring photon flux from bioluminescence (Figure 1). The majority of the devices produce 2D images but a device has been developed that allows a 3D diffuse luminescence tomography (DLIT) that takes into account the scattering and absorption of light in tissue and provides an estimate of the 3D location and brightness of the light-emitting sources (Signore et al., 2010; Thompson et al., 2013). The sensitivity of the technique is influenced by many parameters. One crucial issue is the depth of the luciferase-labeled cells within the body. Upon administration of the luciferin substrate to the transgenic animals, the light emitted by luciferase is able to penetrate tissue depths however light intensity decreases 10-fold each centimeter of tissue depth (Signore et al., 2010). In addition BLI is decreased by pigmentation of fur. Removing fur or alternatively breeding mice into an albino background, can be useful (Signore et al., 2010). In the context of zebrafish models, due to the small size of fishes that reaches 4 cm in length and 0,5 cm of thickness and the absence of fur, difficulties about tissue penetration are slight, owing to the shorter distance through which the emitted light has to travel to reach body surface. The sensitivity of BLI is also influenced by the strength of the promoter used to drive luciferase expression as well as the number of incorporated transgenes per cells. Finally, CCD cameras used are also an important variable that modulates the sensitivity of this technique.

From an ethical point of view, the BLI technology offers significant values. In fact, the assay is performed on the same living animal through a series of non-invasive imaging sessions.

The most important thing is that this technique allows longitudinal experiments to be carried out without sacrificing mice at all times, thus reducing the number of animals needed.

## Transgenic reporter mouse models for BLI

Non-invasive molecular imaging, BLI, is a powerful tool to study single molecular events in time in the physiological setting of a living organism. In the last decade, the application of this technique has been extended from static observation of anatomical structures to dynamic analysis of molecular events. When combined with cancer models, BLI is giving an unprecedented opportunity to investigate in the entire organism the molecular events leading to neoplastic development and progression. Several transgenic mouse models have been developed to visualize cancer growth and cancer-related cellular processes such as proliferation, tumor progression, inflammation and immune processes in living animals by BLI. These mice are not only useful for tracking healthy and disease processes *in vivo*, but also for testing the efficacy of therapeutic compounds. Although some models could be missed, the following list represents an update of transgenic models based on luciferase-dependent imaging generated so far. Tables 1, 2 summarize genetically engineered mouse models (GEMMs) described in this review. In Table 1 are reported GEMMs useful to illuminate cancer-related cellular processes while Table 2 reports GEMMs in which it is possible to image uptake, growth and progression of different cancers.

**Table 1:** List of genetically engineered mouse models useful to illuminate cancer-related processes.

Transgene constructs	Cancer related biological processes	Reference
cycB2 promoter-luciferase	Proliferation	Goeman et al., 2012; Oliva et al., 2013; Spallotta et al., 2013; Rizzi et al., 2015; Principi et al., 2016
ATM promoter-luciferase	DNA damage, heat stress	Gueven et al., 2006
Fusion protein of the entire human p16Ink4a locus and luciferase	Senescence, early steps of transformation	Yamakoshi et al., 2009
_P_16Ink4a promoter-luciferase	Senescence, early steps of transformation	Baker et al., 2011
Endogenous p16INK4a promoter-luciferase	Senescence, early steps of transformation	Burd et al., 2013; Sorrentino et al., 2014
p21 promoter-luciferase	Cell cycle responses upon genotoxic stresses (induced skin tumor)	Ohtani et al., 2007
Endogenous p21 promoter-luciferase	DNA damage	Tinkum et al., 2011; McMahon et al., 2016
Mdm2 promoter-luciferase	P53 activity upon stress treatments	Hamstra et al., 2006
NF-KB promoter-luciferase	Inflammation	Carlsen et al., 2002
Endogenous Cox-2 promoter-luciferase	Inflammation	Ishikawa et al., 2006
IL-ip promoter-luciferase	Inflammation	Li et al., 2008
IL-1 p promoter-luciferase-inflammasome dependent processing site-protein degradation signal (in frame)	Inflammation	Iwawaki et al., 2015
CXCL8 promoter-luciferase	Inflammation	Stellari et al., 2012
c-Rel promoter-luciferase	Inflammation	Yang et al., 2013
Ccl2 promoter-luciferase	Inflammation	Crispo et al., 2013
Nestin promoter-luciferase	Neuroinflammation	Krishnasamy et al., 2017
NF-KB2 promoter-luciferase	Inflammation	Yang et al., 2018
Entire human IL6 locus-luciferase	Inflammation	Hayashi et al., 2015
CAG promoter-renilla luciferase NFAT responsive elements-firefly luciferase	T-cell migration and NFAT-dependent activation	Szyska et al., 2018
Foxp3 promoter-luciferase-diphtheria toxin receptor	Visualization of homeostasis expansion of Foxp3^+^CD4^+^ regulatory T cells	Suffner et al., 2010
CD2 promoter-luciferase-T cell specific enhancer of CD2 (in frame)	T cells monitoring	Chewning et al., 2009
ARR_2_/probasin promoter-luciferase	Androgen receptor activity	Ellwood-Yen et al., 2006
CAG promoter-luciferase-ER folding domain-luciferase (in frame)	Estrogen receptor activity	Sekar et al., 2016

**Table 2:** List of genetically engineered mouse models useful to illuminate cancers.

Luciferase-based GEMMs cross (X) Cancer GEMMs (and/or infections)	Tumors	Reference
POMC promoter-luciferase-Cre recombinase X conditional mutant Rb allele	Pituitary tumor	Vooijs et al., 2002
E2F1 promoter-luciferase X nestin promoter-viral receptor tv infected with avian leukosis virus-based RCAS-PDGFp vector	Glioma	Uhrbom et al., 2004; Momota and Holland, 2005
Prostate specific antigen promoter-luciferase and rat probasin promoter-SV40 large and small T antigens	Prostate cancer	Lyons et al., 2006
P-actin promoter-loxP-GFP-stop codon-loxP-luciferase X cPten^−/−^; probasin promoter-Cre	Prostate adenocarcinoma	Liao et al., 2007
CAG promoter-loxP-stop cassette-loxP-Ezh2-IRES-Luciferase X probasin promoter-Cre	Prostate cancer	Koppens et al., 2017
Endogenous a-fetoprotein promoter-luciferase	Chemical induced hepatocarcinoma	Park et al., 2011
Alpha-fetoprotein promoter-luciferase	Chemical induced hepatocarcinoma	Lu et al., 2011
CAG promoter-loxP-lacZ-neomycin stop codon-loxP-p53^(R172H)^-luciferase-EGFP-KRAS^(G12D)^ infected with Cre-expressing adenovirus with a high tropism for hepatocytes	Hepatocarcinoma	Ju et al., 2015
Insulin promoter-SV40 early region-IRES-luciferase	Pancreatic Pcell tumors and liver metastases	Zumsteg et al., 2010
cycB2 promoter-luciferase X LSL-Kras^ul2D/+;^ X Pdx-1-Cre	Pancreatic ductal adenocarcinoma (PDAC)	de Latouliere et al., 2016
cycB2 promoter-luciferase LSL2Kras^G12D/+;^LSL-Trp53^R172H/+;^ X Pdx-1-Cre	Pancreatic ductal adenocarcinoma (PDAC)	de Latouliere et al., 2016
CAG promoter-loxP-EGFP-stop codon-loxP-E6E7-IRES-luciferase gene Conditional K14-Cre, X LSL-Kras	HPV-positive oral tumor	Zhong et al., 2014
CD19-CherryLuciferase fusion gene(knock-in) X X-MYC	B-Cell lymphoma	Scotto et al., 2012
MMTVpromoter-luciferase X MMTV-PyVT	Mammary tumor	Zagozdzon et al., 2012
MMTV promoter-A16HER2-luciferase	Mammary tumor	Marchini et al., 2011
Knockin into the Gt(ROSA)26Sor locus of loxP-PGK-neo-stop codon-loxP-luciferase fused to the degradation domain of the Hypoxia Inducible Factor 1-a X Bechnl^+/+^; MMTV-NeuT or Beclinl^+/−^,MMTV-NeuT	Mammary tumor	Goldman et al., 2011
Knockin into the hTERT locus of renilla luciferase X TWNT/MTB	Mammary tumor	Jia et al., 2011
ER responsive elements-luciferase	Chemical induced mammary tumor	Vantaggiato et al., 2016
Knockin into the Gt(ROSA)26Sor locus-CMV early promoter/enhancer-loxP-stop cassette-loxP-luciferase-IRES-placental secreted alkaline phosphatase X conditional Pax3-Fkhr	Alveolar rhabdomyosarcoma	Nishijo et al., 2009
β-actin promoter-loxP-stop cassette-loxP-luciferase X conditional oncogenic Kras adenoviral-mediated delivery of Cre recombinase	Lung cancer	Lyons et al., 2003
Endogenous Vegfr3 promoter-EGFP-luciferase	Chemical induced papilloma	Martínez-Corral et al., 2012
HIF-1-dependent promoter-luciferase-HIF-1α X mutant Ha-Ras allele	Chemically induced papilloma and carcinoma	Kadonosono et al., 2011
Tax responsive element-luciferase X granzyme B promoter-Tax oncogene	T-cell leukemia/lymphoma	Rauch et al., 2009b

### Reporter Mice of Cell Growth: Useful Tools for Oncology Research

Progression through each phase of the cell cycle is governed by cyclin/cyclin-dependent kinases (Cdks) complexes. We and others have contributed to demonstrate that the transcription of a plethora of cyclins and cdks, responsible for the cell cycle progression, is driven by the transcription factor NF-Y (Gurtner et al., 2017). Based on the rationale that the activity of NF-Y is restricted *in vitro* to proliferating cells, our group has generated a transgenic reporter mouse, called MITO-Luc (MITO stands for mitosis and Luc for luciferase), in which an NF-Y–dependent promoter controls luciferase expression. The model stems from a decade of research to identify an NF-Y-dependent cyclin B2 minimal promoter driving luciferase expression in all phases of the cell cycle. In these mice luciferase is expressed in each proliferating cell. Using BLI in these mice we visualize areas of physiological cell proliferation as well as regeneration processes in response to injury demonstrating a role of NF-Y activity on hepatocyte proliferation during liver regeneration. MITO-Luc reporter mice would be useful to study the involvement of genes in cell proliferation and aberrant proliferation in disease pathogenesis. They will be also useful to study the effect of new anti/pro-proliferative drugs and assessment of their efficacy both on target and non-target tissues. We have demonstrated that MITO-Luc mouse model is suitable to characterize the effects of small molecules acting on proliferation of bone marrow (BM) and spleen cells in time course experiments in intact animals (Goeman et al., 2012; Oliva et al., 2013; Spallotta et al., 2013; Rizzi et al., 2015; Principi et al., 2016).

Cyclin-Cdk complexes are negatively regulated by Cdk-inhibitors (CKIs) among which p16INK4a and p21Cip1. Apart from their capacity to arrest the cell cycle they have been shown to participate in an increasing number of cellular processes. p16INK4a limits cell-cycle progression and promotes cellular senescence in response to stress such as oncogene activation, while p21Cip1 is mainly involved in growth arrest, quiescence and induction of differentiation.

In 2006 Guaven and colleagues described a mouse model useful to visualize the transcriptional control of ATM, a gene whose mutations lead to a pleiotropic phenotype, including a predisposition to develop malignancies. In these animals the luciferase gene is under the control of the murine Atm promoter. Using this tool the authors demonstrated for the first time that ionizing radiation and heat stress induce ATM promoter in different mouse tissues (Gueven et al., 2006).

To monitor senescence and early steps of transformation transgenic reporter systems driven by fragments of the p16INK4a promoter (Yamakoshi et al., 2009; Baker et al., 2011), have been generated. Yamakoshi and colleagues engineered mice to express a fusion protein of the entire human p16Ink4a locus and luciferase. Baker and colleagues generated transgenic mice carrying a cassette in which a 2,617-bp fragment of the p16Ink4a gene promoter, that is transcriptionally active in senescent cell, drives transcription of luciferase gene. Burd and colleagues targeted luciferase gene into the translational start site of the endogenous p16INK4a locus employing a targeted knock-in strategy concerning in the cloning of luciferase gene into the p16INK4a open reading frame preserving known *cis*-regulatory elements (Burd et al., 2013). In these three systems the authors have been able to monitor senescence and the earliest steps of tumorigenesis suggesting that p16INK4a activation is a common characteristic of early steps of carcinogenesis. Interestingly, the knock-in model has been also employed to study the influence of several environmental agents on aging (Sorrentino et al., 2014).

To monitor cell cycle responses upon genotoxic stresses, reporter models useful to spatio-temporally monitor p21Cip1 activity have been generated. In the model generated by Ohtani et al., 2007, luciferase gene transcription is driving by a p21 promoter fragment. The authors induced skin tumor formation and by time-course BLI experiments they observed that the expression of p21Cip1 oscillates over the time (Ohtani et al., 2007). Later, in 2011 and 2016 two models have been described in which, using a knock-in strategy, the expression of luciferase is placed under the control of endogenous p21 promoter (Tinkum et al., 2011; McMahon et al., 2016). The treatments of these mice with several DNA-damaging agents, including ionizing radiation lead to a temporary light induction. Interestingly, Tinkum and colleagues crossed their mice with p53 null mice, demonstrating that p53 was required for the p21-dependent light. Indeed, both exogenous (Ohtani et al., 2007) and endogenous (Tinkum et al., 2011) p21 promoters are under the control of p53, making these models ideal for monitoring not only p21Cip1 but p53 activation *in vivo*, too.

p53 is a key mediator of cell cycle arrest of stressed cells. Two different reporter models have been generated to track p53 transcriptional activity by BLI. In these mice luciferase gene is transcribed by a p53-dependent promoter, mdm2 and Puma (Hamstra et al., 2006; Briat and Vassaux, 2008). In both papers it is possible to temporal evaluate p53 activity upon stress treatments: ionizing radiation and chemically induced DNA damage, respectively. In view of the major role that p53 plays in normal and transformed cells, these mice represent a powerful tool to predict, map and characterize side effects of drugs helping in the design of new therapeutic strategies.

### Making Tumors Bright

Genetically engineered mouse models of human cancer are a useful tool to investigate molecular mechanisms of tumorigenesis (Tuveson and Hanahan, 2011). The utility of GEMMs in preclinical research may improve even further when they are combined with non-invasive bio-imaging techniques. GEMMs carrying bioimaging reporter systems that tag specific cellular processes or molecular events observed in human cancers are useful tools for early detection of cancer as well as for understanding of cancer initiation, immune system roles, tumor angiogenesis, invasion and cancer therapy. In the past years several different GEMMs have been generated in which cancer growth can be followed by BLI. These models represent several big killer cancers among which pancreatic, prostate, brain, breast and liver cancer.

The pioneer example of bright tumor in mice is that of pituitary tumor development. Vooijs and colleagues (Vooijs et al., 2002) took advantage of animals carrying one conditional mutated Rb allele. They crossed these mice with mice expressing Cre recombinase and luciferase gene under the control of a pituitary specific promoter. These mice endogenously develop pituitary bright tumors. These mice are a powerful tool to study cancer prevention and treatment using anticancer agents that interfere with the Rb pathway.

To measure brain tumor growth non-invasively, Ef-luc transgenic mice expressing luciferase gene under the control of the E2F1 promoter have been crossed GEMM of human gliomas. The E2F1 promoter is autoregulatory during late G1 and S phase of the cell cycle being inhibited by E2F-RB during early G1. Ef-luc mouse line crossed with the N–tv-a mouse strain expressing the viral receptor tv-a from the nestin promoter. Upon infection with a viral vector expressing RCAS-PDGFB, oligodendrogliomas have been induced in these mice and the authors were able to follow development of these tumors by BLI. This system has been used to follow early stages of brain tumorigenesis and to examine where tumors initiate (Uhrbom et al., 2004; Momota and Holland, 2005).

Several murine models have been developed to real-time monitoring of prostate cancer growth based on the use of a specific prostate promoter. In one model luciferase is under the control of the PSA promoter, whose activity is confined to the epithelial cells of the prostate. In these animals the expression of SV40 large T antigene is targeted to prostate using the rat probasin promoter. In these mice prostate lesions rise progressing from hyperplasia through to high-grade lesions that eventually metastasize. The authors demonstrated that these mice are useful to follow the tumor response to androgen ablation in a long timeframe (Lyons et al., 2006). In another example a composite promoter fragment from PSA promoter has been used to generate the PSA-E2/P-luc transgenic mice. In these mice the luciferase is mainly expressed in the ventral prostate lobes. This model, when bred unto the TRAMP prostate cancer background allows not only the uptake but also the progression of distant soft-tissue metastasis of prostate cancer (Seethammagari et al., 2006). On the basis of these data it is reasonable to assume that if the transgenic mouse model PSA-Luc were crossed with transgenic models that develop prostate cancer it would be possible to visulalize the onset, growth and progression of prostate cancer through BLI. The other model take advantage of the Cre/loxP technology, inactivating Pten alleles and activating luciferase and GFP expression in the prostate epithelial cells. Interestingly, although Cre expression is under an androgen-dependent promoter, once the genes undergo recombination neither the Pten allele nor luciferase requires androgen for expression thus making this model useful for monitoring growth, regression, or relapse of cancer irrespective of hormonal manipulations (Liao et al., 2007). On the other end, a model useful to image androgen receptor signaling is the transgenic mouse ARR2 Pb-Lux that expresses luciferase specifically in the prostate in an androgen-dependent fashion (Ellwood-Yen et al., 2006). Recently, a mouse model has been generated in which it is possible to visualize prostate tumor growth and metastatic progression over time. In this mouse model the Polycomb Group protein EZH2, whose overexpression is associated with poor prognosis in prostate cancer, and luciferase gene are transcribed together. The prostate-specific expression of the transgene is mediated by crossing of these mice with Probasin-Cre carrying mice (Koppens et al., 2017).

In 2011 Lu and colleagues described the generation of a knock in mice in which a thymidine kinase and luciferase reporter genes were placed under the transcriptional control of the endogenous alpha-fetoprotein (AFP) promoter. In the same year a transgenic mouse expressing luciferase under control of AFP promoter has been described (Park et al., 2011). Both models have been demonstrated to be useful to monitor chemical induced hepatocarcinoma by BLIs (Lu et al., 2011). Recently, a GEMM has been developed in which, following Cre-mediated DNA excision, the two oncogenes p53^R172H^ and KRAS^G12D^, are expressed in a single open reading frame with luciferase and GFP genes. In these mice, systemic injection of Cre encoding adenovirus induce the expression of the four transgenes in the liver leading to the development of bright liver tumors The bioluminescent signals correlates with tumor sizes, demonstrating the utility of this model for BLI of liver tumor development (Ju et al., 2015).

With the purpose to monitor pancreatic T-cell tumors non-invasively by BLI, Zumsteg and colleagues in the 2010 developed mice in which the expression of a bicistronic mRNA coding for large T antigen and luciferase occurs specifically in β-cells of Langerhans islets. In these mice the authors were able to illuminate tumor progression as well as lymph node and liver metastases (Zumsteg et al., 2010).

Genetically engineered mouse models designed to recapitulate genetic and pathologic aspects of pancreatic ductal adenocarcinoma (PDAC) are the LSL-Kras^G12D/+^;Pdx-1-Cre (KC) and LSL-Kras^G12D/+^;LSL-Trp^53R172H/+^;Pdx-1-Cre (KPC) mice, in which the Cre-recombinase, transcribed by the pancreas-specific Pdx-1 promoter, leads to the expression of oncogenic mutant for of Kras alone or in combination with a mutant p53 protein, respectively (Hingorani et al., 2003, 2005). Our group has recently developed and characterized two novel mouse models, MITO-Luc-KC (MKC) and MITO-Luc-KPC (MKPC), obtained through intercross of KC and KPC with our MITO-Luc mouse model, engineered to express the luciferase reporter gene in cells undergoing active proliferation. In these mice we have had the opportunity to follow PDAC evolution in the living animal in a time frame process. Altogether, *in vivo* and *ex vivo* analyses demonstrate that MKC and MKPC mouse models are powerful tools for visualizing PDAC development in terms of proliferation in the entire living animal in a spatio-temporal manner. Most notably, these results also demonstrate that, in these mouse models, it is possible to identify early steps of pancreatic carcinogenesis non-invasively in living animals in which proliferation events take place before tumor appearance (de Latouliere et al., 2016). From a pharmacological point of view, this opens the possibility to design therapeutic protocols with a more precise timing than those using the tumor palpability as a starting point, a parameter of low specificity and sensitivity.

To track responses to small molecules, Zhong and collegues developed and HPV-positive oral tumor mouse model in which HPV oncogenes E6, E7, mutant kras as well as a luciferase reporter (iHPV-Luc) are conditionally express in the epithelial cells upon a tamoxifen-regulated Cre recombinase system. In these transgenic mice tamoxifen treatment resulted in oral tumor development the development of which can be easily monitored by BLI (Zhong et al., 2014).

To achieve bioluminescence in B-cell lymphoma Scotto and colleagues have developed a transgenic mouse carrying CherryLuciferase fusion protein targeted to one allele of the CD19 locus crossed with GEMM of an aggressive lymphoma due to a chromosomal translocation of the c-myc gene (Scotto et al., 2012).

For BLI mammary tumor visualization and monitoring, a mouse strain expressing luciferase and Polyoma Virus middle T antigen in mammary glands under control of MMTV promoter (Zagozdzon et al., 2012) has been generated. Similarly, Marchini and colleagues, to examine the ability of Δ16HER2, a splice variant of HER2 gene, to induce transformation of mammary epithelium and to monitor Δ16HER2-mediated tumorigenesis in alive mice, developed a mouse strain in which transcription of luciferase and Δ16HER2 are under control of MMTV promoter (Marchini et al., 2011). Goldman and colleagues in the 2011, developed a transgenic mouse model in which regions of hypoxia are imaged via BLI thanks to the expression of the oxygen-dependent degradation domain of the Hypoxia Inducible Factor 1-α gene in frame with luciferase gene. These mice have been cross-breed with transgenic mice developing mammary tumors (MMTV-neu and Beclin1 +/−), and tumor growth were longitudinally tracked over several weeks and in response to treatments (Goldman et al., 2011). Based on this proof-of-principle the authors hypothesized that this GEMM could serve as a platform for non-invasive imaging of solid tumors in mice. An interesting model has been generated useful to image the transcriptional regulation of human TERT expression. These mice carry a 160-kb transgenic bacterial artificial chromosome containing the human TERT locus and Renilla luciferase cassette downstream human TERT promoter (Jia et al., 2011). This model has been crossed with mice overexpressing Wnt1 oncogene by a Dox-dependent promoter activated via mammary-specific expression of reverse tetracycline transactivator. Mammary adenocarcinomas developed consistently in female mice and imaging of human TERT transcription has been followed in mammary gland during tumorigenesis. To determine the dynamics of estrogen receptor activity, GEMM expressing a luciferase reporter gene under the control of estrogen receptor responding elements has been generated. This study has been the pioneer in the field of transgenic luciferase-reporter animals models and, using young mice before sex hormones production and/or ovariectomized adult mice, it highlights, for the first time, the importance of hormone-independent activation of the estrogen receptor (Ciana et al., 2003). This model has been later used to demonstrate the applicability of BLI to the study of ER signaling during breast transformation. The authors induced sporadic mammary cancers in these mice with DMBA (9,10-dimethyl 1,2-benzanthracene) and imaged estrogen receptors activity by BLI from early stages to palpable tumors in the breast (Vantaggiato et al., 2016). Recently, a GEMM expressing an ERα folding-biosensor has been developed. In these mice it is possible to evaluate the impact of diverse estrogenic ligands and to study the estrogen induced carcinogenesis *in vivo* (Sekar et al., 2016).

Luciferase activity measured by BLI in entire living animals allow a spatio-temporal imaging of its expression. However, it is a semi-quantitative measure being possible only a relative measure between the different individuals and/or the different treatments. Nishijo et al. (2009) developed a bicistronic Cre/LoxP reporter mouse strain in which semiquantitative, spatially expression of luciferase is coupled with quantitative expression of a serum biomarker, the human placental secreted alkaline phosphatase (SeAP). These mice have been crossed with an inducible Pax7-CreER mouse reporter line, allowing a muscle specific expression of luciferase and SeAP and with a mouse model of rhabdomyosarcoma. In this contest they investigate correlations between tumor volume, luciferase signal and serum SeAP levels, demonstrating that they have an higher precision than the traditional pre-clinical models (Nishijo et al., 2009).

To image spontaneously arising tumor burden, Lyons and colleagues developed a ubiquitously expressed conditional (Cre/Lox) luciferase transgene. In these mice it is possible to visualize cells that have undergone Cre-mediated recombination in alive animals. The mice have been crossed with transgenic mice in which a conditional oncogenic Kras expressed in lung epithelial cells induces non-small cell lung cancer and the authors demonstrate that, in this contest, longitudinal non-invasive visualization of lung tumorigenesis by BLI is feasible (Lyons et al., 2003).

An interest GEMM is represented by the Vegfr3 mouse model, where an EGFP-luciferase fusion protein, is expressed under transcriptional control of the endogenous Vegfr3 promoter. In these mice Martinez-Corral and colleagues were able to track tumor-induced lymphangiogenesis in the context of papillomas induced by dimethylbenzanthracene/12-O-tetradecanoylphorbol-13-acetate (DMBA/TPA) (Martínez-Corral et al., 2012). Similar to the GEMM developed by Goldman and collegues, an HRE/ODD-luciferase mouse model consisting of a HIF-1-dependent promoter and a cDNA encoding luciferase fused to the sequence of the ODD domain of human HIF-1α has been developed (Kadonosono et al., 2011). These mice were crossed with mice carrying a Ha-Ras mutated allele. which show a higher sensitivity to carcinogens than conventional mice. Treatment of these mice with N-methyl-N-nitrosourea (MNU), leads to bioluminescent tissues contained papillomas and carcinomas.

### Imaging Cancer-Associated Inflammation and Immune Response in Mice

Among different transgenic models developed to spatio-temporally image inflammation in living animals, the most used is the one generated by Carlsen et al., 2002. In these mice expression of luciferase is driven by NF-kB response elements. Playing NF-KB a major role in physiological and pathological inflammation, these mice are a very useful tool to visualize inflammation in intact animals. They were used to image inflammation in any body district associated with several inputs among them pro- and anti-inflammatory treatments including stressors (Carlsen et al., 2004; Vykhovanets et al., 2008; Vykhovanets et al., 2012; Young et al., 2014), b-islet and cardiac transplantation (Roth et al., 2006; Ma et al., 2008), myocardial infarction (Tillmanns et al., 2006), Escherichia Coli infection of mammary glands (Notebaert et al., 2008), autoimmunity (Zangani et al., 2009) and high fat diet (Vykhovanets et al., 2011). Other tools to image chronic and acute inflammation are two knock-in mice in which luciferase gene is cloned in the endogenous cyclooxygenase-2 locus (Ishikawa et al., 2006). In other transgenic mouse lines useful to image inflammation, transcription of luciferase gene is driven by promoters of genes involved in inflammation processes, such as a Smad2/3-dependent promoter (Lin et al., 2005), human IL-1beta (Li et al., 2008; Iwawaki et al., 2015), CXCL8 (Stellari et al., 2012), c-Rel (Yang et al., 2013) ccl20 (Crispo et al., 2013), nestin (Krishnasamy et al., 2017) and NFkb2 (Yang et al., 2018) promoters. A sensitive GEMM useful to monitor inflammation has been generated integrating luciferase reporter gene in a bacterial artificial chromosome containing sequences of the human interleukin 6 gene (Hayashi et al., 2015). In all these animal models, systemic inflammation has been monitored in a variety of healthy and damaged tissues. However, any of these interest models has yet been studied in the contest of cancer-related processes.

Few studies visualize inflammation processes related to cancer. One interest study by Rauch and colleague have employed a mouse model of spontaneous lymphoma in which inflammation associated to malignant transformation is coupled with light emission. They crossed transgenic mice in which luciferase transcription is regulated by HTLV-1 LTR promoter with mice expressing Tax oncogene under the human granzyme B promoter in activated T cells and natural killer cells. These mice develop leukemia and lymphoma and the authors were able to discover an already unknown inflammation step preceding tumorigenesis (Rauch et al., 2009b). Using this model the authors were also able to demonstrate that inflammatory stimulus leads to the development of lymphoma (Rauch et al., 2009a).

Although bioimaging of immune response is a challenging item and many efforts have been made to date to visualize *in vivo* the immune response to antigens relatively few transgenic animal models have been produced. To study adoptive T-cell therapy efficacy in the presence of large solid tumors a transgenic mouse models that allow monitoring T-cell activation in response to cancer has been generated. In this model renilla luciferase is under control of a constitutive promoter while firefly luciferase transcription is driven by the NFAT promoter thus allowing the concomitant analysis of T-cell migration and NFAT-dependent activation. The T-cells isolated from these mice are useful for longitudinal studies have been employed to study the kinetics of T-cell activation upon immune reconstitution of myelo ablated hosts in the presence of two tumor models (Szyska et al., 2018). Suffner and colleagues in 2010 generated transgenic animals in which it is possible to visualize homeostasis expansion of Foxp3^+^CD4^+^ regulatory T cells (Tregs). These cells play a major role in maintaining self-tolerance and limiting immune responses to pathogens. In these mice the promoter of Foxp3 gene transcribes luciferase and the diphtheria toxin receptor. By BLI the authors demonstrated that Tregs were mainly located in lymphoid organs. Treg depletion upon diphtheria toxin treatment was also monitored by BLI, and longitudinal studies showed that the Treg compartment was recovered to its original size due to homeostatic proliferation of the remaining Tregs (Suffner et al., 2010). To study T cell dynamics following antigen encounter, a GEMM has been generated using a human CD2 mini-gene to drive luciferase transcription on T cell compartment. To real time image antigen-specific CD4^+^ T cell kinetics, the authors crossed these mice with OVA-specific CD4 TCR transgenic mice and analyzed CD4^+^ T cell antigen-specific responses. They were able in this system to compare kinetics and magnitude of clonal expansion/contraction in lymph nodes and tissue sites of antigen injections (Chewning et al., 2009). Although the two last models have not been used to follow cancer–associated immune response, they definitely represent useful tools for future studies in immuno-oncology research.

## Real-Time Imaging of Gene Expression: Light Insights From Zebrafish

Despite 450 million years of evolutionary distance, cell and molecular pathways that govern signaling, proliferation, differentiation, and apoptosis are highly conserved between human and zebrafish, thus Zebrafish models represent a fundamental tool to improve treatments of malignant disease, as an intermediate experimental step between cell culture based assays and human clinical trials. Contrary to what has been published for mouse models, there are still few transgenic zebrafish models that exploit BLI by driving the expression of luciferase reporter gene under the control of a regulatory sequence or embedded on endogenous loci. Due to the optical transparency, defective or pathological phenotypes can be analyzed in the whole-mount embryos using fluorescent proteins that allows the study of the disease processes. On the other hand, the detection of fluorescent proteins in living adult animals is particularly tricky, due to the presence of the no-transparent skin of adult zebrafish that avoids to detect signals from an internal organ and restricts the observation on tissues near the body surface. Multiple advantages of bioluminescence overcome fluorescence detection in living adult animals among which the lack of background noise signal and the fact that the wavelength of the light emitted from luciferase-expressing cells is enough to penetrate all tissues in small animals.

Up today the BLI technology has been mainly applied to zebrafish tumor xenograft model, using luciferase labeled cancer cells to track tumor growth *in vivo* and to test the efficacy of antitumor and antiangiogenic compounds (Letrado et al., 2018). Noteworthy Chen’s group in the 2013, for the first time, has applied BLI methodology in a transgenic zebrafish model. They found that luciferase activity in adult zebrafish can be reproducibly monitored in tissues of freely swimming animals in a non-invasive manner. Although melanin absorbs light, the photons emitted from inner tissues do not show signal attenuation by the melanophores (Chen et al., 2013). Both ubiquitous and tissue-specific luciferase-based transgenic lines has been described. In the ubiquitous lines, ubiquitin or β-actin2 promoters drive widespread luciferase expression. On the contrary, in the tissue specific lines, cryaa or cmlc2 promoters drive luciferase expression specifically in the lens and cardiomyocytes, respectively. Interestingly, in the cmlc2-luc line, authors were able to demonstrate that luciferase-dependent imaging is useful to estimate muscle quantity in the inner organ, the heart, and to longitudinally follow cardiac regeneration after injury.

A second example of BLI methodology applied to a transgenic zebrafish model comes from the paper of Astuti and colleagues in the 2017. The authors generated a zebrafish strain that ubiquitously expressed luciferase gene under control of the ubiquitin promoter. Hematopoietic stem/progenitor cells have been collected from marrow contained in the kidney of adult zebrafish of this strain and it has been transplanted in an irradiated non luciferase expressing zebrafish strain. In this model system it is possible to track by BLI, hematopoietic cell homing function, immediately after the hematopoietic cell transplantation and using this model they have been able to demonstrate a positive role of ergosterol, a vitamin D2 precursor, in the homing of hematopoietic cells (Astuti et al., 2017).

In the same year, another zebrafish model has been generated in which an NF-κB promoter fragment drives expression of luciferase and GFP. Inflammation treatments of these animals, such as TNFα, induces a dose-dependent luciferase signal in live transgenic embryos demonstrating that this models is a valuable tool for studying NF-κB signaling in a spatiotemporal resolution manner (Kuri et al., 2017).

## Drawbacks of Luciferase-Mediated Imaging

As described so far, the use of BLI in preclinical studies certainly has many advantages. However, when interpreting the results, it is important to take into account several shortcomings. BLI is a semiquantitative method and all results are relative to each other that’s why it’s not easy to standardize the experiments. The BLI signal could not be directly proportional to cell number since both endogenous and exogenous factors can impact the various steps of the luciferase/substrate reaction and could lead to misinterpretations. It is important to have in mind that the amount of luciferases, luciferin and cofactors such as ATP can be different in different cells/body districts (Fan and Wood, 2007). Half-life and stability of different luciferases can vary between few hours to several days. In case of use of secreted luciferases it’s important to take into account that some circulating components of blood and urine can also affect the enzyme stability (Czupryna and Tsourkas, 2011). The technique is restricted to small animals. Indeed, the depth and the location of the luciferase-expressing cells in the body can impact on the BLI signal. The administration route of the substrate is also critical for BLI. After i.p. or i.v. injections d-luciferin has different biodistribution being more homogeneous among tissues when the substrate is administered i.v. However, i.p administration yielded a prolonged organ uptake of the substrate (Berger et al., 2008). Light quenching is mediated by pigmented molecules (e.g., hemoglobin) and depends to the depth of the source of the signal. Hair and fur can scatter and attenuate light signal. The black fur color of the animals can attenuate the light and the signal is lower in black compared to white mice (Edinger et al., 2002). Although the high sensitivity of BLI is due to its low background, light emission by substrates such as coelenterazine, in the absence of the enzyme, can increase the luminescence background.

## Conclusion and Future Perspectives

It has been evident for over a century that cancer is a systemic disease but the participation of the host macro-environment in tumor development and progression is only beginning to be appreciated. Recently, several emerging evidences highlight the temporal and spatial activation of hematopoiesis and immune response during cancer progression. Indeed cancer tissue- and lymphoid-related circuits promote reciprocal interplay with unexpected complexity. For example, it has been described that luminal breast cancer (LBC) cells establish a systemic macro-environment that supports outgrowth of otherwise-indolent disseminated tumors. LBCs secrete cytokines that activate BMCs (Kuznetsov, 2012). This cascade results in growth of adenocarcinoma and is abolished when BMCs activation is inhibited by an anti-inflammatory compound such as aspirin. These findings highlight the systemic macro-environment as an important component of disease progression (Becker, 2014). However, the crosstalk between macro-environment and tissue target of disease is still not yet widely known and there are still important unanswered questions. The lack of this knowledge are primarily due to the lack of human and animal models in which it is possible to follow tumor evolution in a time frame process.

BLI reporter animals are going to revolutionize the way biological processes, among which cancer, can be studied. Signals are generated with a fast kinetics, enabling the visualization of molecular events in real time, not only on the target organs but in all body districts allowing the visualization of the communicative reprogramming occurring in the entire animal. The non-invasive nature of the model allows investigations of physiological events from embryos to adult animals, as well as longitudinal studies of disease progression and/or drug response. The ability to monitor individual animals throughout longitudinal studies without sacrificing them significantly reduce the number of animals required in each experiment. It will also be possible to detect even sligth changes due to variability among individuals. Although they are already giving important results on basic and applied cancer research, we can anticipate that in the future they will be increasingly used in translational cancer research, finding application among the others in drug discovery development and toxicology.

## Author Contributions

IM, LdL, AG, and GP conceived and designed the review. GP wrote the manuscript. All authors read and approved the final manuscript.

## Conflict of Interest Statement

The authors declare that the research was conducted in the absence of any commercial or financial relationships that could be construed as a potential conflict of interest.
